# Caffeic Acid on Metabolic Syndrome: A Review

**DOI:** 10.3390/molecules26185490

**Published:** 2021-09-09

**Authors:** Nellysha Namela Muhammad Abdul Kadar, Fairus Ahmad, Seong Lin Teoh, Mohamad Fairuz Yahaya

**Affiliations:** 1 Department of Anatomy, Faculty of Medicine, Universiti Kebangsaan Malaysia Medical Centre, Cheras, Kuala Lumpur 56000, Malaysia; nellysha.namela@ums.edu.my (N.N.M.A.K.); fairusahmad@ukm.edu.my (F.A.); teohseonglin@ukm.edu.my (S.L.T.); 2 Department of Biomedical Sciences and Therapeutics, Faculty of Medicine and Health Sciences, Universiti Malaysia Sabah, Kota Kinabalu 88400, Malaysia

**Keywords:** caffeic acid, metabolic syndrome, phenolic compound, obesity, dyslipidemia, hyperglycemia, hypertension

## Abstract

Metabolic syndrome (MetS) is a constellation of risk factors that may lead to a more sinister disease. Raised blood pressure, dyslipidemia in the form of elevated triglycerides and lowered high-density lipoprotein cholesterol, raised fasting glucose, and central obesity are the risk factors that could lead to full-blown diabetes, heart disease, and many others. With increasing sedentary lifestyles, coupled with the current COVID-19 pandemic, the numbers of people affected with MetS will be expected to grow in the coming years. While keeping these factors checked with the polypharmacy available currently, there is no single strategy that can halt or minimize the effect of MetS to patients. This opens the door for a more natural way of controlling the disease. Caffeic acid (CA) is a phytonutrient belonging to the flavonoids that can be found in abundance in plants, fruits, and vegetables. CA possesses a wide range of beneficial properties from antioxidant, immunomodulatory, antimicrobial, neuroprotective, antianxiolytic, antiproliferative, and anti-inflammatory activities. This review discusses the current discovery of the effect of CA against MetS.

## 1. Introduction

Metabolic syndrome (MetS) has affected almost one fifth of the adult population and increases the risk of cardiovascular disease, type-2 diabetes, and all-cause mortality compared to a healthy person [1]. In Asia, Malaysia is recognized as one of the countries that has a high MetS prevalence [2]. MetS is a complication of the modern lifestyle that includes overeating and underactivity [3]. With the current COVID-19 pandemic situation and increasing state of sedentary lifestyle, the numbers are bound to be more than the expected figures in the coming years [4].

The current definition of MetS still uses the Harmonized Criteria that state that abnormal findings of 3 out of 5 of the following risk factors would qualify a person of having MetS: raised blood pressure, dyslipidemia (raised triglycerides (TG) and lowered high-density lipoprotein cholesterol), raised fasting glucose, and central obesity [5,6]. These components have the ability to precede into cardiac dysfunction, but together, they can also cause an additional risk to morbidity and mortality [7]. Although MetS has been collectively accepted as an alarming condition, the clinical world has yet to mutually agree on a uniform terminology and diagnostic criteria. This is mainly due to the adversity of genetic predisposition, diet history, and physical, geographical, and endocrinal attributes that together take part in forming this intricate syndrome [8]. One of the causes of MetS is the increase in oxidative stress and chronic inflammation. In many instances, it has been shown that an antioxidant imbalance may play a role in its development where there is an overproduction of reactive oxygen species (ROS) and nitrogen (RNS) species that can react with virtually all biomolecules, causing oxidative damage [9,10]. Similarly, human studies have also shown that MetS is associated with oxidative stress and a proinflammatory state that comes with a high antioxidant defense in the peripheral blood mononuclear cells assumed to be derived from a pre-activation state of human cells [11].

Although obesity and insulin resistance remain at the root of MetS pathogenesis, other factors such as chronic stress and dysregulation of the hypothalamic–pituitary–adrenal axis and autonomic nervous system, increased cellular oxidative stress, renin–angiotensin–aldosterone activity, and intrinsic tissue glucocorticoid reaction, as well as the newly discovered miRNAs, have been identified to play roles in this condition [12,13].

At the core of many pathological diseases, including MetS, an increase in ROS has played a crucial element that can be tipped over with the aid of a longstanding diet comprising antioxidants [14]. Reactive species are essential signaling molecules that are involved in nearly every physiological activity, from cell division to metabolic regulation. They modulate the activity of biomolecules, and redox-sensitive transcription factors activate a cell’s adaptive endogenous response, including antioxidant defense. The degree of reactive species production and neutralization that are tightly associated with oxidative metabolism determines the redox homeostasis of cells and their surroundings. Setting the redox states of cells is critical in both health and disorders such as MetS [15]. The question is, which antioxidant and at what aliquot would be the optimum elixir to shorten the period in combating the specific diseases. 

The research world has, for many years, focused on a more natural approach toward combatting human diseases. Synthetic medications have slowly proven its downside over years of pharmacological use. Polypharmacy in the treatment of MetS has become a substantial healthcare burden due to adverse drug reactions, morbidity, and cost [16]. One of the phytonutrient compounds that caught the attention of researchers were the flavonoids. These are a very diverse group of polyphenolic compounds that consists of a benzo-γ-pyrone and can be found in several parts of a plant. They are classified as plant secondary metabolites having a polyphenolic structure [17,18]. These compounds, which can be found in abundance in the Mediterranean diet, has increasingly shown a beneficial effect in maintaining cardiometabolic and cardiovascular health, which, in turn, reduces the risks of MetS development. This positive impression may be due to the diets that are high in polyphenolic antioxidant content derived from vegetables, grapes, and olive oils [19]. Similarly, treatment with naringin, a type of glycoside flavonoid, has been reported to reverse MetS by reducing visceral obesity, blood glucose, blood pressure, and lipid profile [20].

In this review, we discuss a phenolic compound found in many herbs, caffeic acid (CA), or its chemical name 3,4-dihydroxycinnamic acid, which belongs to a group called phenolic compounds, which are a naturally occurring chemical structure found abundantly in fruits and vegetables [21,22]. 

## 2. Caffeic Acid as a Phenolic Compound

Phenolic compounds provide protection against noncommunicable diseases not only by their means of antioxidant activity but also by regulating a variety of cellular processes at different levels, including enzyme inhibition, modification of gene expression, and protein phosphorylation [23]. An increase in phenolic compounds can alter their health benefits [24]. There are over 8000 phenolic compounds that can be classified into two main groups: flavonoids and nonflavonoids. Flavonoids contain a phenyl benzopyran skeleton: two phenyl rings joined through a heterocyclic pyran ring. Nonflavonoids, on the other hand, are mostly smaller and simpler in comparison to flavonoids [17]. 

**Figure 1:**
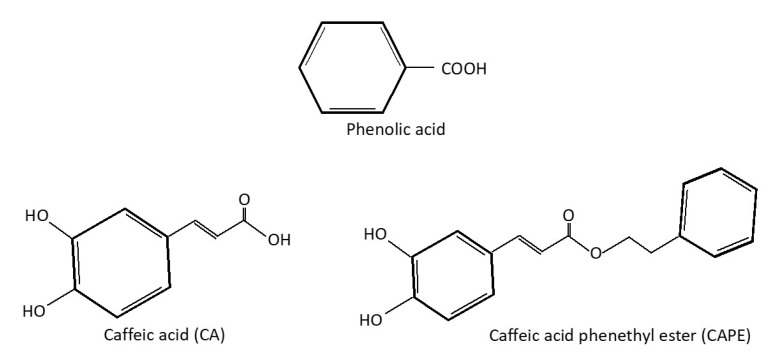
Chemical structure of PA, CA, and CAPE.

Phenolic acids (PAs) are a group of nonflavonoid phenolic compounds that contain a single phenyl group substituted by a carboxylic group and one or more hydroxyl (OH) groups [25]. PAs are further divided according to the length of the chain that contains the carboxylic group into: hydroxybenzoic acids, hydroxycinnamic acids, and hydroxyphenyl acids. The group hydroxycinnamic acid has a C6-C3 (phenylpropanoid) basic skeleton. Hydroxy derivatives of cinnamic acid are more effective as an antioxidant than the hydroxyl derivatives of benzoic acid as the presence of a CH_2_ = CH-COOH group in the cinnamic acids ensures a greater antioxidant capacity than the COOH group in benzoic acid (Figure 1). One of the major hydroxycinnamic acids is CA [26,27,28].

CA is found in coffee, honey, potatoes, berries, herbs, and vegetables such as olives, Swiss chard, and carrot [29]. In vitro and in vivo studies have shown that CA not only possesses antioxidant capacity but also has immunomodulatory [30], antimicrobial [31], neuroprotective, antianxiolytic [32], antiproliferative, and anti-inflammatory activities [33], and has shown to improve inflammation and oxidative stress in chronic metabolic diseases. Besides the therapeutic potentials of CA, studies have also shown that the pure form of CA has the availability to be absorbed in the intestines and form subsequent interactions with the target tissue [34]. This solidifies the potential of using CA as an oral route of administration as an appealing choice for a phytonutrient.

CA has also been found in Gelam honey and stingless bee honey through HPLC analysis [35,36]. The antioxidant capability of CA is due to its ability to scavenge ROS, including O2−, OH−, and H_2_O_2_ [37]. CA has shown to be an effective ABTS, DPPH, and superoxide anion radical scavenger, with a total reducing power and metal chelates on ferrous ion activities, in comparison to other standard antioxidant compounds such as BHA, BHT, alpha-tocopherol, and trolox in different in vitro antioxidant assays [38]. Multiple factors influence PA efficacy in vivo, including the amount of consumed chemical, whether it is absorbed or metabolized, its plasma or tissue concentrations, PA type and dosage, and synergistic effects [39].

Besides pure CA, its derivatives in the form of caffeic acid phenyl ester (CAPE) and caffeic acid phenylethyl amide (CAPA) have also been found to have a therapeutic effect against MetS. However, CAPA and CAPE are less stable in its form compared to CA [40]. CAPE is an active component of the propolis substance and has been known for its anti-inflammatory, antioxidant, and anti-cancer effects [41]. The following section discusses the effects of CA and its derivatives on different components of MetS.

## 3. CA vs. Obesity

Obesity is a condition where excess body fat accumulates either due to the enlargement of lipids in existing adipocytes (hypertrophy), or through an increase in the number of adipocytes (hyperplasia) [42]. Adipose tissue in the human body functions as an energy storage system, an endocrine gland, and a heat productor (nonshivering thermogenesis) [43]. In healthy slender individuals, adipocytes are smaller, more insulin-sensitive, and secretes anti-inflammatory mediators such as adiponectin, IL-10, IL-4, IL-13, IL-1 receptor agonist (IL-1Ra), apelin, and transforming growth factor beta (TGFβ). In contrast, the adipocytes of an obese individual are enlarged and infiltrated by a large number of pro-inflammatory M1 macrophages that secrete pro-inflammatory cytokines such as TNFα, IL-6, visfatin, leptin, MCP-1, Ang-II, and plasminogen activator inhibitor-1 [44]. With the surplus of these pro-inflammatory compounds within the obese adipocyte, they are often referred to be in a state of inflammation. This state of chronic low-grade activation of the innate immune system is critical in the pathophysiology of obesity and MetS [45].

Visceral fat is localized within the abdomen and is metabolically active with the constant release of free fatty acids into the portal circulation [46]. In a state of caloric excess, the hypertrophied adipocytes will secrete adipokines that result in the increment of additional pre-adipocytes that will later mature. However, this compensatory act reaches its threshold and causes fat accumulation in the visceral depots. The accumulation and distribution of the fat depots play a key role in forming metabolic complications. A metabolically healthy obese individual that remains insulin-sensitive and displays a normal metabolic and hormonal profile and is physically different compares to a metabolically unhealthy obese person through their higher abdominal circumference measurement [47]. 

Metabolic changes in obesity are associated with a persistent low-grade inflammatory state that impairs energy homeostasis and glucose metabolism [44,48]. The c-Jun N-terminal kinase (JNK) and the nuclear factor-kappa B (NF-κB) signaling pathways contribute to inflammation and play a key role in obesity, insulin-resistance, and in regulating the expression of proinflammatory molecules [49]. Zhang and colleagues found that CA was able to exert anti-inflammatory effects in dextran sulfate sodium-induced colitis mice, showing a significantly suppressed secretion of IL-6 and TNFα and colonic infiltration of CD3+ T cells, CD177+ neutrophils, and F4/80+ macrophages through the activation of the NF-κB signaling pathway. Their study concluded that CA was able to amend the colonic pathology and inflammation, indirectly contributing toward reducing obesity [50]. 

Obesity may also be associated with adipocyte necrosis, which could be the start of a pro-inflammatory response. Adipocytes grow hypertrophic when their caloric intake and energy expenditure increase, which has been linked to cell hypoxia and death. These hypertrophic adipocytes will subsequently start secreting TNFα in small amounts, resulting in a chemotactic response that draws macrophages [48]. An in vitro study using adipose stem cells (ASCs) showed that CAPE had the ability to inverse the effects of high glucose and lipopolysaccharide exposure. Through this study, they found that CAPE treatment was able to restore the functions of adipocytes by increasing the adiponectin and peroxisome proliferator-activated receptor gamma (PPARγ), resulting in the reduction in pro-inflammatory factors [51]. CA also acts on adipogenesis by reducing intracellular lipid accumulation in an in vitro model [52]. 

Increasing evidence has shown that gut microbiota plays a role in the development of obesity and MetS through the modulation of energy absorption, and subsequently influences glucose and lipid metabolism [53,54]. It was recently postulated that gut microbiota producing t10,c12-conjugated linoleic acid induced lipogenesis [8]. Dietary polyphenols have been found to promote the growth of beneficial bacteria while inhibiting pathogenic bacteria [55]. In an in vivo study to determine the anti-obesity effect of CA, high-fat-diet (HFD)-induced mice were seen to have a positive effect after being given a daily dose of 50 mg/kg CA for a span of 12 weeks. The researchers noted a significant reduction in body weight and fat accumulation, increases in energy expenditure and beneficial gut bacteria (i.e., Muribaculaceae), and a decrease in pathogenic bacteriae (i.e., Lachnospiraceae) [56]. 

In another study, HFDs in nonalcoholic fatty liver disease (NAFLD)-induced mice were used to demonstrate the effectiveness of CA treatment and its effects toward the gut microbiota. CA was able to significantly reduce the body weight of the HFD-fed mice and attenuated the expression of lipogenesis-related protein expression (Srebp1, Fas, Acc, and Scd1) in the liver. It was concluded that CA exerted protective effects on the NAFLD mice by inhibiting gut dysbiosis, pro-inflammatory LPS release, and subsequent lipid synthesis [57]. 

## 4. CA vs. Hyperglycemia and Insulin Resistance

One of the primary causes of metabolic and endocrine abnormalities, as well as cellular damage in afflicted tissue, is hyperglycemia-related oxidative stress [15]. Nutrient-induced toxicity due to overnutrition may lead to insulin-resistance in tissues such as the heart and the skeletal muscle, which normally responds to insulin for glucose uptake [58]. Insulin resistance is a condition where the tissues use their adaptive mechanism to avoid toxic nutrient overload [59]. Over time, insulin resistance will cause an increase in fasting glucose and reduced insulin-mediated glucose clearance. Eventually, hyperinsulinemia will occur as a negative feedback from the target cells, signaling inadequate insulin response, and, in turn, the pancreatic β-cells will produce more insulin. The prolonged inability to correct the state of insulin resistance will eventually give rise to hyperglycemia and type 2 diabetes [60]. 

CA is found to increase insulin sensitivity through the reduction in proinflammatory cytokines and increase in adiponectins under the hyperglycemic state [51]. In a study that used MetS diet-induced rats, where it caused increases in BMI and abdominal circumference, blood glucose, triglycerides, and LDLc, and lowered the HDLc, the group that received a dose of 40 mg/kg oral gavage of CA daily for 6 weeks showcased a significant reduction in serum leptin, adiponectin, insulin, TNF-a, IL-6, and IL-8. The study showed that CA had the highest superoxide dismutase (SOD), catalase, and glutathione peroxidase antioxidant enzymes in the liver after 4 weeks of CA administration in comparison to ferulic acid, gallic acid, and protocatechuic acid under the same doses [61]. This suggests that the scavenging activity as a result of CA administration shows the most promising effectivity amongst the listed phenolic acids that protect against hyperglycemic damages.

Nasry et al. investigated the role of pioglitazone (a synthetic PPARγ agonist that causes a decrease in insulin resistance) on HFD-induced-MetS rats, and CA was able to show promising results. There was a significant reduction in insulin resistance, fasting blood glucose, and fasting serum insulin with an increase of insulin sensitivity and β cell function. CA also reduces the nitric oxide (NO) liver contents to almost half of those of the HFD-induced MetS rats [62]. This shows the efficacy of CA as scavenging activity toward correcting the insulin resistance through the reduction in oxidative stress caused by the HFD.

CA also suppresses the hepatic glucose output by enhancing its utilization and inhibiting overproduction [63]. This can be seen by the increase in glucokinase activity through an increase in its mRNA expression and glycogen content. It was also found to simultaneously lower the G6Pase and phosphoenolpyruvate carboxykinase activities together with their respective mRNA expressions, along with a decline in the GLUT2 expression in the liver [63]. 

CA methyl and ethyl esters exert antidiabetic activities in insulin-responsive cells through insulin-independent mechanisms involving AMPK and adipogenic factors [64]. A 2-week treatment of CAPA toward streptozotocin and diet-induced diabetic mice were able to protect them against hepatic inflammation and glucose intolerance associated with the NF-κB-mediated induction of inflammatory cytokines and the increase in the expression of antioxidant protein. HepG2 cell models were then used to further investigate CAPA’s ability. They were able to show that CAPA was able to ameliorate TNFα-induced pIKKα/β expression and prevent TG accumulation in H_2_O_2_-treated HepG2 cells [40]. These findings strengthen the belief that chronic oral administration of CAPA is able to protect against MetS.

Stress-induced inflammation may cause the development of insulin resistance [65,66,67]. Stress activates the hypothalamic–pituitary–adrenal axis, renin–angiotensin system pathway, and sympathoadrenal system, all of which are involved in the production of pro-inflammatory cytokines, resulting in the negative downregulation of insulin signaling by either phosphorylating insulin resistance serine residues or inhibiting Akt, resulting in insulin resistance. CA given to chronic restraint stress-induced insulin-resistance mice showed to reduce fasting blood sugar, systemic inflammation, and oxidative stress, and improve insulin sensitivity [68].

## 5. CA vs. Dyslipidemia

Dyslipidemia is described as an abnormal level of circulating lipids. It has been acknowledged that dyslipidaemia increases the risk of cardiovascular disease development [69]. This condition may be of primary cause (genetic) or secondary (diet, drugs, chronic diseases, and metabolic disorders, including MetS). Dyslipidemia is detected through a biochemical analysis of fasting lipid profile, which consists of TG, total cholesterol (TC), high-density lipoprotein cholesterol (HDL-c), low-density lipoprotein cholesterol (LDL-c), and non-HDL-c. Dyslipidemia is diagnosed when there is an increased concentration of TG, TC, LDL-c, and non-HDL-c, along with a decreased level of HDL-c [70]. 

Free fatty acids (FFA) are abundantly released in an obese body due to the increase in the adipose tissue mass. FFA causes an increase in the synthesis of glucose and TG in the liver, as well as an increase in VLDL secretion. This occurs together with the reduction in HDL-C and increased density of LDL [71]. CA has shown improvement in the serum lipid profile, serum liver biomarker enzymes, and hepatic tissue architecture to normal in HFD-induced hyperlipidemic rat models by showing antihyperlipidemic and hepatoprotective activities. CA was found able to reduce the levels of endoplasmic reticulum stress markers in the liver after a HFD obese induction [72]. Besides CA’s ability to revert dyslipidemia by reducing TG and TC, studies have shown that CA was able to revert hepatic steatosis in the long run [49,73,74,75]. In a recent in vivo study, a 12 week CA supplementation on HFD obese mice revealed that CA was able to reduce body weight and fat accumulation together with readings of improved lipid profile with an increased HDL [56]. This suggests that CA’s ability to impair the formation of bad white fat tissue could subsequently reduce FFA production, thereby showing its hepatoprotective ability. 

CA is capable of providing a TG-lowering, anticoagulatory, antioxidative, and anti-inflammatory protection for the cardiac tissue and also downregulating the TNF-α and monocyte chemoattractant protein-1 mRNA expression in the kidney of diet-induced diabetic rats [76]. Studies on diet-induced hypercholesterolemic rats by Agunloye and Oboh compared the modulatory properties of CA and chlorogenic acid, proving that CA was a better candidate in ameliorating the pathological condition. They also tested two different dosages of the drug (10 mg/kg and 15 mg/kg of CA) and concluded the lipid-lowering effects were more effective at larger doses [77].

It is possible that an excessive amount of oxidative stress and/or inflammation can convert circulating LDL and HDL particles into oxidized LDL (oxLDL) and oxidized HDL particles (oxHDL). OxLDL and oxHDL both stay longer in the bloodstream due to their impaired interaction with their specific receptors. Their diminished clearance and imbalance of lipid profile ultimately contributes to the onset of atherosclerosis [19]. CA is thought to prevent atherosclerosis by lowering the functional and structural changes in the arteries [78]. This has been demonstrated by its ability to inhibit thrombogenic thromboxane A2 (TXA2) production together with other platelet-aggregating molecules [79,80]. CA also downregulated platelet-activating molecules such as COX-1, calcium ions, and P-selectin and upregulated platelet-inhibiting molecules such as cAMP and cGMP, resulting in an inhibition toward thrombogenic processes [81].

## 6. CA vs. Hypertension

Almost 80% of the individuals with MetS suffer from hypertension. Evidence concurred that 65–75% of the risk factor for primary hypertension is contributed by obesity and excess weight gain [82]. Besides, insulin resistance has also been linked to hypertension as insulin is able to cross the blood–brain barrier and subsequently activate the systemic nervous system, in addition to its ability to upregulate the angiotensin II (AT-II) receptor and reduce NO [60]. NO is one of the most important ROS in the cardiovascular system. ROS are produced by NO synthase enzymatically, and they act as a prototype endothelial-derived vasodilator [83].

Nω-Nitro-L-arginine-methyl ester (L-NAME) is a well-known active inhibitor of NO production in the nerves and the endothelial cell. A study using L-NAME-induced hypertensive rats showed that a combination of caffeine and CA was able to reduce the systolic BP. A decrease in ACE and arginase activity coupled with high NO and low MDA levels might be associated with their antihypertensive effects [84,85]. In another study using CAPE against the high-fructose corn syrup diet-induced vascular damage in rats, blood pressure values were significantly reduced after a two-week intraperitoneal injection with CA derivative. This study also noted that CAPE has the ability to correct the reduced levels of endothelial NO synthase levels caused by the high-fructose corn syrup diet [73]. 

According to a more recent study, CA has a favorable effect on the vascular function and blood pressure stabilization. In this study, male SERCA2a knockout mice and its wild-type were surgically implanted with mini osmotic pumps filled with AT-II solutions and fed with a normal diet of 0.05% CA in drinking water. CA significantly attenuated the AT-II-induced increase in blood pressure reading in the wildtype mice but showed no hypotensive effect to the SERCA2a knockout mice. This suggests that the CA might act by activating the SERCA2a on the primary vascular smooth muscle cells [86].

**Figure 2:**
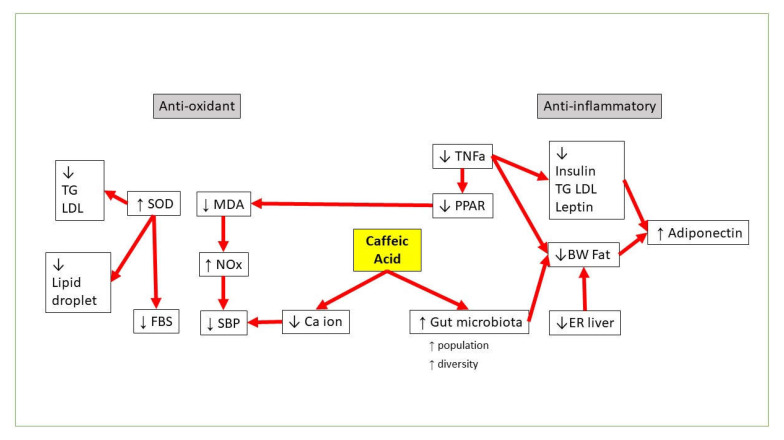
Proposed CA pathways against MetS.

CA has also been reported to be a potent antihypertensive agent and has been confirmed to have a nontoxic manifestation [87,88]. Agunloye and Oboh’s in vitro study revealed that CA was capable of inhibiting key enzymes associated with hypertension that includes E-NTPDase, 5′-ectonucleotidase, ADA, ACE, arginase, and AChE. This study suggested that CA targets specific enzymes associated with hypertension [89]. Decreased ACE and arginase activity, as well as high NO and low MDA levels, might be associated with their antihypertensive effects [77]. The summary for MetS studies related to CA can be found in Table 1, whereas the proposed pathway for CA against MetS can be found in Figure 2.

## 7. Conclusions

There has been enormous progress in understanding the effect of CA through retrospective research. Strong evidence of the ability of CA to reverse the MetS effects through the reduction in inflammatory markers such as TNFα coupled with reduced oxidative stress parameters have guided researchers to a more proteomic and metabolomic approach. Besides the singular usage of CA, studies of using CA as an enhancer together with more commonly used drugs have surfaced. Through this review, we can conclude that CA holds strong potential to be used as MetS management by its anti-obesity, antidiabetic, hypolipidemic, and hypotensive activities. During the course of drafting this manuscript, we identified a substantial gap in which the wealth of knowledge about CA is limited to findings in animal models or cell lines. Further studies in the form of a clinical trial or a population cohort study would further strengthen the beneficial effect of CA on MetS.

## Figures and Tables

**Table 1:** MetS studies related to CA.

Pathological Induction/State	Dose of CA or Its Derivates and Administration Route	Duration of Treatment	Observations	Reference
Diet-induced MetS with HFD in male Wistar rats	40 mg/kg via oral gavage	6 weeks	Reduced: —Insulin —HOMA-IR —Leptin —TNFα —IL-6 —IL-8 —Total cholesterol, TG,VLDLc, LDLc,HDLc Increased: —Adiponectin	[61]
Diet-induced hypercholesterolemic rats	10 and 15 mg/kg	21 days	Reduced: —Total cholesterol —TG —LDL —HDL (With dose 15 mg/kg showing better results) Increased: —Plasma and heart SOD activity	[77]
Nω-Nitro-L-argininge-methylester (L-NAME)-induced hypertensive in male Wistar rats	5 mg/kg and 25 mg/kg via oral gavage	20 days	Reduced: —SBP —MDA Increased: —ACE activity —NOx level	[85]
Surgically implanted mini osmotic pumps filled with Ang II solution in wild type mice and SERCA2a knockout mice	0.05% CA in drinking water	8 weeks	CA was able to: —Relax mesenteric artery —Smooth norepinephrine-induced vasoconstriction —Reduced intracellular Ca^2+^ ions —Bind to SERCA forming strong hydrogen bonds —Significantly attenuated AngII-induced hypertension. However, CA failed to do so in SERCA2a knockout mice	[86]
HFD obesity-induced C57BL/6J mice	50 mg/kg via oral gavage	12 weeks	Reduced serum insulin	[56]
Alloxan-induced type-1 diabetic in Swiss albino mice	50 mg/kg intraperitoneal injection	7 days	Protective effects on liver and kidneys Hypoglycemic and hypolipidemic properties.	[75]
STZ-induced diabetic male Wistar rats	10 and 50 mg/kg via oral gavage (diluted in canola oil)	30 days	Reduced —FBS —oxidative stress parameters (lipid peroxidation, reactive species production, protein oxidation, and MPO activity).	[90]
STZ-induced diabetic rats	orally	5 weeks	Increased: —serum insulin level —GSH, CAT, and SOD levels Reduced: —Blood glucose level Histologically seen normal islet morphology in CA administered diabetic rats.	[91]
STZ and high-fat high-fructose-diet-induced CD1 (ICR) mice	10 mg/kg/day of CAPA orally	2 weeks	Reduced —Body weight increase —Plasma retinol binding protein 4 (RBP4) —Adiponectin level —TNFα in liver Preserved glucose tolerance Prevented glucose intolerance Preserved basal coronary flow	[40]
Insulin-resistant adipocytes ASCs exposed to high glucose levels			Decreased lipid droplets and radical oxygen species formation. Increased insulin sensitivity (showed reduction in pro-inflammatory cytokines level and increased adiponectins).	[51]
HFD inducing NAFLD in C57BL/6J mice	0.08% or 0.16% CA added to pellet diet	8 weeks	Reduced body weight in both concentrations. Positively altered the community compositional structure of gut microbiota.	[57]
Non-insulin-dependent DM (NIDDM) and insulin-resistant (IR) mice models	15 and 30 mg/kg CAPE dissolved in PEG-400 given via oral gavage.	5 weeks	Improved: —Insulin sensitivity —Hyperlipidemia —Peroxisome-proliferator-activated receptor-α (PPAR-α) —TNFα —Glucose consumption —Glucose uptake —Glycogen content —Oxidative stress level —Decreased level of glucose-6-phosphotase expression (G6Pase).	[49]
HFD-induced obesity in mice	50 mg/kg/day orally	10 weeks	Reduced: —Body weight —Liver weight —Liver lipid accumulation —Levels of ER stress markers in the liver Improved glucose intolerance and insulin sensitivity.	[72]
High fructose corn syrup- induced vascular dysfunction in Sprague Dawley rats	50 mmol/kg intraperitoneal injection	2 weeks	Reduced SBP Increased NO synthase production. Significant reduction in TC and LDL. No significant change to HDL nor TG.	[73]
Chronic restraint stress-induced insulin resistance in LACA mice	5 and 10 mg/kg intraperitoneal injections	30 days	Reduces: —Fasting blood sugar —Systemic inflammation —Oxidative stress —Improved insulin sensitivity	[68]
HFD-induced MetS in C57 mice	A combination of ferulic acid (50 mg/kg/day) with CA 0.9 mg/kg/day via subcutaneous injection	40 days	Prevents obesity. Reverts hyperglycemia. Reverts dyslipidemia. Reverts hepatic steatosis.	[74]
High-fat-diet and STZ-induced diabetic male Wistar rats	40 mg/kg via oral gavage	8 weeks	Improved albumin excretion by kidneys. Improved blood glucose Reduced renal mesangial matrix extension. CA results were seen better in reversing the diabetic nephropathy in comparison to prevention.	[92]
L-NAME-induced Sprague Dawley rats	50 µmol/kg/day intraperitoneally	14 days	Kidney tissue analysis shows that CA was: —Unable to preserve PON1 activity —Unable to reduce NF-κB significantly	[93]
Hyperlipidemic Wistar Albino	20 mg/kg/day	30 days	Significantly reduced: —Total cholesterol —TG —HDL-c	[94]
